# Optimizing the Cow Manure-Straw Ratio to Promote Organic Matter Humification: Insights from Three-Dimensional Fluorescence Spectroscopy

**DOI:** 10.3390/plants15050729

**Published:** 2026-02-27

**Authors:** Liangshi Hao, Yan Li, Shuang Wang, Yarun Wang, Yu Hu, Yangyang Xia, Zhixin Qi, Hongsheng Gao, Dan Wei, Wei Li

**Affiliations:** 1 College of Resources and Environment, Northeast Agricultural University, Harbin 150030, China; haoliangshi_24@163.com (L.H.); yangyangxia2025@163.com (Y.X.); qz0329x@163.com (Z.Q.); 2 Key Laboratory of Black Soil Protection and Utilization, Heilongjiang Fertilizer Engineering Technology Research Center, Heilongjiang Academy of Black Soil Protection and Utilization, Harbin 150086, China; li.yan622@163.com (Y.L.); wangshuang0726@163.com (S.W.); ghs6837@163.com (H.G.); 3 College of Resources and Environment, Shanxi Agricultural University, Taiyuan 030800, China; 18636177620@163.com; 4 Soil Health Laboratory in Shanxi Province, Institute of Eco-Environment and Industrial Technology, Shanxi Agricultural University, Taiyuan 030800, China; 5 Institute of Plant Nutrition, Resources and Environment, Beijing Academy of Agricultural and Forestry Sciences, Beijing 100097, China; huyu0805@126.com

**Keywords:** dissolved organic matter, humification, fluorescence spectroscopy, material ratio, composting environment

## Abstract

Straw and cattle manure are common agricultural wastes, and their composting plays a critical role in regional nutrient cycling and organic carbon management. During composting, the structural evolution and humification processes of dissolved organic matter (DOM), fulvic acid (FA), and humic acid (HA) are regulated by environmental factors such as temperature and pH. However, systematic studies on the multi-component fluorescence characteristics of DOM in straw–manure systems and their coupling with environmental variables remain limited. In this study, maize straw and cattle manure were used as raw materials, with four mixing ratios (T1–T4: 2:8, 4:6, 6:4, and 8:2), to investigate the effects of raw material proportions on the structural evolution of DOM, fulvic acid (FA), and humic acid (HA) during composting. Three-dimensional fluorescence spectroscopy combined with parallel factor analysis (EEMs-PARAFAC) was applied to characterize organic components, their transformation patterns, and their relationships with environmental factors. The EEMs-PARAFAC identified 3, 2, and 3 components for DOM, FA, and HA, respectively. Moderate straw–cow manure ratios (T2 and T3) maintained high microbial activity while promoting humic-like component accumulation and FA-to-HA conversion. Fluorescence indices indicated mixed substrate-derived and microbial sources for DOM, predominantly microbial origins for FA, and a shift in HA from substrate-derived to mixed sources. Overall, humification remained low (humification coefficient < 1.5), reflecting an early composting stage. Mantel analysis and partial least squares path modelling (PLS-PM) revealed temperature as the dominant factor associated with HA formation, whereas an alkaline pH inhibited humification. These findings clarify how substrate ratios regulate humification via environmental microhabitats, providing a theoretical basis for optimizing straw–manure co-composting and enhancing compost stability and soil carbon sequestration.

## 1. Introduction

The composting of agricultural organic waste is a key approach to effectively use resources and produce fertilizer. Straw and livestock manure, as common agricultural waste compounds, can be used to address agricultural waste pollution and facilitate resource recycling, promoting economic development and ecological conservation. The complex transformation of humus during the composting of agricultural organic waste is a key process in compost humification and carbon stabilization [1]. Compared with regulation strategies based on additives, aeration, or microbial inoculation, adjusting the straw–cattle manure ratio represents a more fundamental, source-oriented approach in composting. Differences in carbon quality, nitrogen availability, and substrate structure between straw and manure mean that their mixing ratio directly exerts a relatively higher influence on the initial C/N balance, substrate degradability, and the supply of humification precursors. By regulating organic matter transformation from labile components to humic substances at the source, this approach provides mechanistic insights into DOM evolution and humification. Dissolved organic matter (DOM), as the most reactive organic component in composting systems, is most critical for microbial metabolism and chemical reactions. Its structural evolution directly determines the humification rate and organic matter stability [2]. Although DOM only accounts for a small fraction of the total organic carbon in compost, it substantially impacts the decomposition and humification of organic matter as well as carbon transformation and stabilization [3]. In this sense, elucidating the dynamic characteristics of DOM and its regulatory mechanisms is crucial for understanding humification. Fulvic acid (FA), the water-soluble fraction of humic substances, represents a humified component within dissolved organic matter (DOM), which also contains a wide range of non-humified organic compounds. By contrast, humic acid (HA) is insoluble under acidic conditions and represents a chemically distinct humic fraction rather than a component of DOM [4]. Whilst FA is a crucial precursor for humus formation, HA reflects the degree of system maturation and structural stabilization [5]. The novelty of applying parallel factor analysis in this study lies not in the technique itself, but in its integrative application to DOM, FA, and HA within a unified analytical framework. By simultaneously resolving fluorescent components in these three operational fractions, this approach treats DOM, FA, and HA as successive and interrelated forms along the humification continuum rather than as isolated entities. This enables a direct comparison of component composition and dynamics across fractions, providing mechanistic insights into the transformation pathways and redistribution of organic matter during composting.

Humification has traditionally been described as a progressive transformation of organic residues into complex and chemically stable humic substances through oxidative polymerization and polycondensation reactions, leading to increasingly aromatic and high-molecular-weight structures, as summarized in classical works such as Stevenson. However, more recent perspectives have challenged the strictly macromolecular view of humic substances. The proposed supramolecular model suggests that humus substances are composed of relatively small heterogeneous molecules, which are bound together by weak intermolecular forces rather than through the covalent polymerization of large molecules [6], indicating that humification involves multiple concurrent processes, including partial polymerization, oxidative coupling, structural rearrangement, and progressive enrichment in condensed aromatic moieties [7]. Therefore, humification is now widely understood as a dynamic continuum of molecular transformation rather than a single defined polymerization pathway. Spectroscopic methods such as UV–visible and fluorescence spectroscopy have been validated in seminal work for evaluating the chemical nature and humification degree of humic substances from soils and composts [8]. In recent years, excitation–emission matrix fluorescence (EEMs) combined with parallel factor analysis (PARAFAC) has been widely applied to characterize the DOM structure. It enables a molecular-level identification of fluorescent components from different sources and types, revealing the degradation and polymerization pathways of organic matter [9,10]. Studies across compost and soil systems have demonstrated that the relative abundances and compositional evolution of FA and HA components, as resolved by EEM–PARAFAC analysis, are closely associated with the degree of humification and compost maturity [11]. Studies on various compost and soil systems have demonstrated that the protein- and humic-like components and their relative abundances, as identified via EEM–PARAFAC analysis, can reflect humification and compost maturity [12]. Partial Least Squares Path Modelling (PLS-PM) provides a unified framework for constructing and testing hypothesized causal pathways among variables, distinguishing between direct effects and indirect effects. By integrating multiple observed indicators into latent variables, PLS-PM can quantify the relative contributions of different environmental factors and organic matter characteristics to the humification process, thereby clarifying dominant drivers and mechanistic pathways in composting systems. Optical parameters such as the fluorescence index (FI), the biogenic index (BIX), and the humification index (HIX) can reflect the origin and transformation stage of DOM: increases in FI and BIX predominantly occur during the early stage dominated by microbial activity, whereas an elevated HIX indicates enhanced aromaticity and polymerization [13]. Therefore, by integrating EEM-PARAFAC components with the aforementioned fluorescence indices, the transformation of DOM from proteinaceous to humic forms can be traced at the molecular level, enabling the evaluation of the humification degree and the environmental behavior of compost products.

Straw and cattle manure are the main agricultural organic waste products in Northeast China, and their effective treatment as carbon and nitrogen sources is significant for composting systems [14]. In the straw–cow manure composite composting system, temperature, pH, and aeration conditions are the key factors influencing DOM [15]. Temperature regulates the rate of enzymatic reactions, thereby influencing the formation of aromatic intermediates and the oxidation polymerization reaction [16]. The pH determines the ionization state of active groups such as carboxyl and phenolic hydroxyl groups, impacting the direction and rate of condensation reactions between DOM molecules [17]. High temperatures and weakly acidic environments promote the conversion of FA to HA, thereby enhancing humus accumulation and material stabilization [18]. The effects of different material ratios and their relatively stronger influence on strategies on DOM structure and humification have been extensively investigated in sludge and organic solid waste composting systems. However, most studies have focused on single raw materials or individual enhancement approaches [19]. Systematic studies on the multi-component fluorescence characteristics of DOM in the straw–cow manure system and its relationships with environmental variables such as temperature and pH are scarce.

In this study, we used corn stover and cattle manure as raw materials, designing composting treatments with varying material ratios to systematically investigate the effects of mixing proportions on the structural evolution of key components, i.e., DOM, FA, and HA, during composting. By employing EEMs-PARAFAC and fluorescence indices (FI, BIX, HIX), we analyzed the fluorescence characteristics and transformation patterns of DOM, FA, and HA, along with their coupling relationships with key environmental factors such as temperature and pH. By doing so, we elucidated the mechanism by which environmental conditions regulate the humification pathways of organic matter in raw waste through precise raw material ratios. We established optimized strategies for combining straw and manure resources to provide a theoretical foundation for enhancing the stability and fertilizing potential of agricultural waste compost products. Briefly, the main novelties include (i) regulating the composting process by adjusting the straw–cattle manure ratio rather than relying on additives, aeration optimization, or microbial inoculation, thereby providing a source-oriented perspective on how raw material composition intrinsically governs DOM evolution and humification pathways; (ii) the simultaneous application of EEM–PARAFAC analysis to dissolved organic matter (DOM), fulvic acid (FA), and humic acid (HA) within a unified analytical framework, enabling a comparative evaluation of fluorescent components across different operational fractions along the humification continuum; (iii) the combined interpretation of fluorescence indices (FI, BIX, and HIX) for DOM, FA, and HA to distinguish substrate-derived and microbially derived contributions and to elucidate the dynamic nature of humus formation during composting; and (iv) the use of partial least squares path modeling to quantify both direct and indirect effects among environmental conditions, DOM characteristics, and humification indicators, thereby providing mechanistic insights beyond simple correlation analysis.

This study was designed to address the following hypotheses/questions:

(i) whether adjusting the straw–cattle manure ratio intrinsically regulates DOM composition and its transformation trajectories during composting by altering substrate availability and biodegradability;
(ii) whether the evolution of fluorescent components differs systematically among dissolved organic matter (DOM), fulvic acid (FA), and humic acid (HA), reflecting a continuous and fraction-dependent humification process;
(iii) whether environmental conditions indirectly influence humification efficiency by mediating DOM structural characteristics rather than through simple direct effects.

## 2. Results

### 2.1. Changes in Temperature, pH, and Organic Matter During Composting

**Figure 1:**
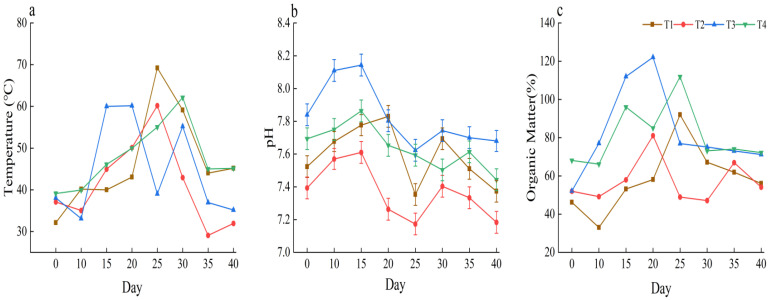
(**a**) Changes in temperature throughout the composting period, (**b**) Changes in pH throughout the composting period, (**c**) Changes in organic matter throughout the composting period.

The composting temperatures across all treatments increased first and then decreased (Figure 1a). Peak temperatures of 69 °C and 60 °C, in T1 and T2, respectively, occurred on day 25. In T3, the peak temperature of 60 °C was reached at 15 days, whereas in T4, the peak temperature of 62 °C was reached at 30 days. This reflects the impacts of raw material ratios on the heating rate and the duration of the high-temperature phase, with a higher proportion of cattle manure potentially stimulating early microbial activity and accelerating an increase in temperature. The pH increased first and then decreased throughout composting (Figure 1b), ranging from 7.17 to 7.69. The initial increase in pH is mainly a result of ammonia nitrogen release during ammonification, and the subsequent decline is associated with organic acid accumulation and nitrification. Notably, the pH of treatment T2 was significantly lower than that of T3 (by 0.5), indicating that differences in the composition significantly influenced the acid-base balance of the system. Similarly, the organic matter content first increased and then decreased (Figure 1c). After 40 days, organic matter had increased by 56.8% to 96.4% compared to the initial stage (10 days). The organic matter content in treatment T1 was significantly higher than that in T3 (by 25.5%; *p* < 0.05), indicating that the content of highly humified organic matter will increase and accumulate continuously, while most of the initial un-decomposed organic matter was mineralized into carbon dioxide and lost accordingly.

### 2.2. Parallel Factor Analysis of Fluorescent Components in Fermented Materials

**Figure 2:**
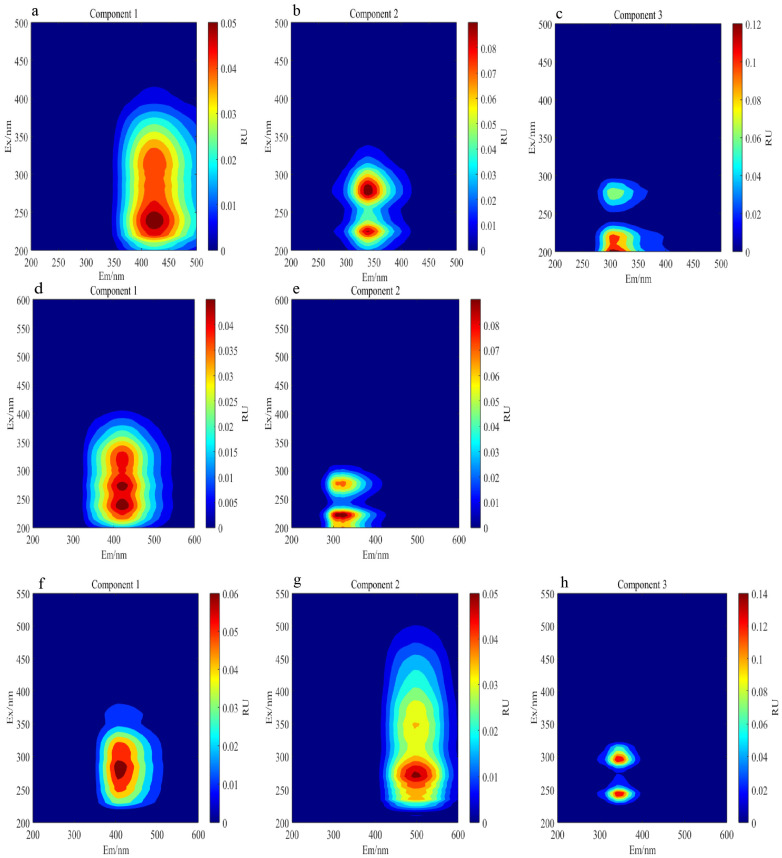
Three DOM (dissolved organic matter) fluorescence components identified by parallel factor analysis (**a**–**c**), two FA (fulvic acid) fluorescent components identified by parallel factor analysis (**d**,**e**), three HA (humic acid) fluorescent components identified by parallel factor analysis (**f**–**h**).

Three fluorescent components were identified in the DOM of the compost (Figure 2): C1 (Ex/Em = 240/425 nm) represents a humic-like component associated with microbial metabolism and humification processes [20]. The compound C2 (Ex/Em = 275 (280)/340 nm) is the tryptophan-like component associated with microbial activity [21], whereas C3 (Ex/Em = 200/305 nm) is a tyrosine-like component belonging to the class of tyrosine-like substances and considered a degradation product of peptides/proteins [22]. For FA, two components were identified (Figure 2): C1 (Ex/Em = 240/420 nm), a terrestrial humic substance associated with photochemically mediated transformation products or photoresistant degradation processes [23], and C2 (Ex/Em = 220/320 nm), a tryptophan-like component closely associated with microbial activity and metabolic processes [24]. For HA, three components were identified (Figure 2): C1 (Ex/Em = 285/405 nm) represents humic-like substances, correlated with organic matter accumulation and associated with elevated organic matter levels in fermented materials [25], and C2 (Ex/Em = 270/495 nm) represents long-wave humic substances, which can attenuate subsequent rapid decomposition processes and promote organic carbon stabilization [26]. The compound C3 (Ex/Em = 245 (295)/340 nm) represents microbial-derived humic substances [27].

### 2.3. Changes in the Relative Abundances of Fluorescent Components (Fmax)

**Figure 3:**
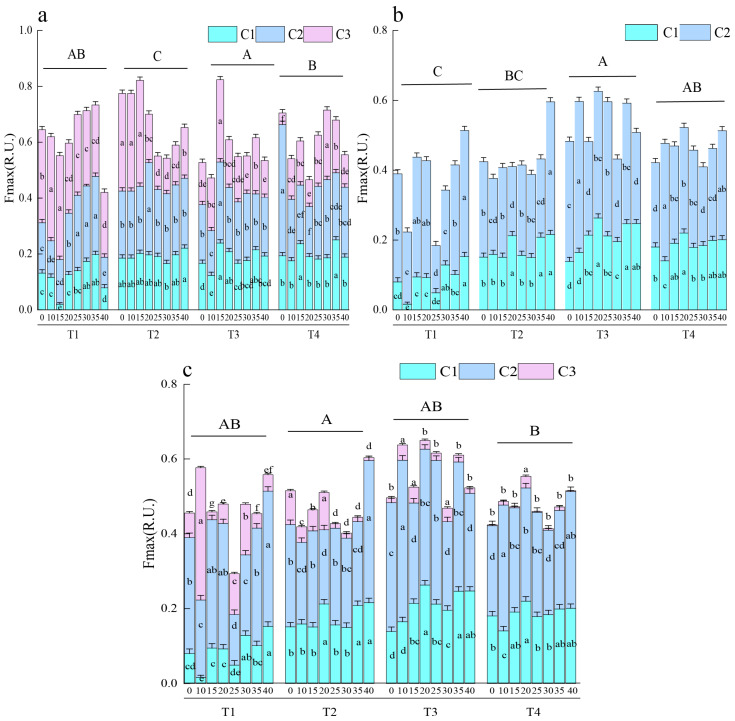
Significance analysis of maximum fluorescence intensity of (**a**) DOM, (**b**) FA, and (**c**) HA across different treatments and sampling days. Lowercase letters indicate significant differences (*p* < 0.05) among sampling times within the same fluorescent component. Uppercase letters indicate significant differences (*p* < 0.05) in overall levels among treatments.

The Fmax value serves as a semi-quantitative indicator of the relative abundances of fluorescent components during composting. In this study, the Fmax range for DOM was 0.43–0.82. The values decreased with composting in T1, T2, and T4 and increased in T3. This indicates that the appropriate ratio of straw to cattle manure in T3 created a more favorable microenvironment for humification, promoting the accumulation of fluorescent components in DOM. The Fmax values of FA (0.18–0.62) and HA (0.10–0.24) increased during composting, indicating the gradual enrichment of humic components via the synthesis of macromolecules from small molecules. Treatment T1 exhibited significantly lower Fmax values in FA than T3 and T4 (*p* < 0.05), with decreases by 4.36% and 3.52%, respectively. Conversely, T2 showed significantly higher Fmax values in HA than in T4 (*p* < 0.05), with an increase of 2.15%. This demonstrates that the composting material ratio regulates the pathways of humic substance formation (Figure 3).

### 2.4. Fluorescence Index Characteristics and Their Significance in Humification Indication

**Figure 4:**
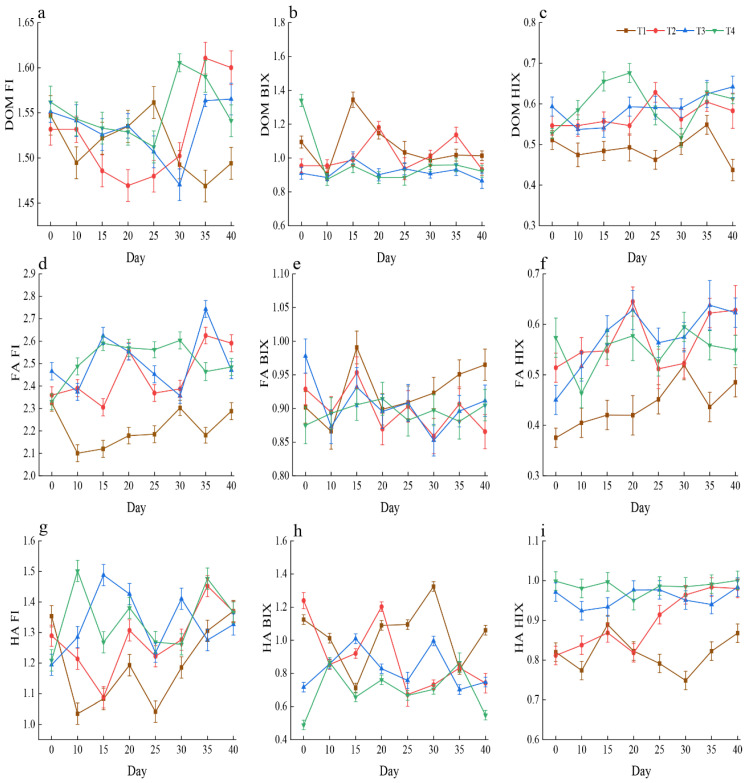
Changes in the fluorescence index of (**a**–**c**) DOM, (**d**–**f**) FA, and (**g**–**i**) HA across different treatments and sampling days.

Using three-dimensional fluorescence spectroscopy, the fluorescence indices FI, BIX, and HIX were applied to characterize the optical properties, compositional evolution, and humification-related features of DOM, FA, and HA throughout the composting process. These indices were used as qualitative indicators of organic matter transformation rather than for strict source attribution. Throughout composting, the FI values of DOM ranged from 1.47 to 1.62, reflecting a mixed fluorescence signature shaped by both substrate-derived organic matter and microbial processing. This intermediate range suggests that DOM functioned as a transitional pool undergoing continuous structural transformation rather than representing a distinct source category. The FI values of FA were consistently high (2.10–2.74), indicating strong protein-like and microbially processed fluorescence features. These characteristics suggest that FA predominantly represented low-molecular-weight and relatively labile organic components formed during active organic matter transformation, consistent with its role as an intermediate fraction during composting. In contrast, FI values of HA exhibited greater temporal variability. During the initial composting stage, FI values ranged from 1.04 to 1.50, with some treatments displaying relatively low values, reflecting the dominance of substrate-associated aromatic structures at early stages. As composting progressed, FI values increased to approximately 1.4–1.5, indicating an increasing incorporation of microbially transformed components into the HA fraction. These observations confirm that HA is not a static humic pool but a dynamic organic fraction that undergoes continuous structural modifications during composting. The BIX values of DOM ranged from 0.87 to 1.34. During the early composting stage (0–10 days), the BIX values in all treatments consistently exceeded 0.8, indicating a strong contribution of newly generated, biologically derived organic components. With composting progression, the BIX values gradually declined, suggesting a reduction in newly produced labile substrates and a progressive shift toward more aromatized and structurally complex materials within DOM. The BIX values of FA remained relatively stable throughout the composting process (0.85–1.00), with no significant differences among treatments. This stability indicates that FA consistently functioned as an actively transformed organic fraction closely linked to microbial metabolism. In contrast, the BIX values of HA showed a wider range (0.49–1.24). During the early composting stage, lower BIX values in some treatments indicated limited incorporation of freshly produced components, whereas, during the middle to late stages (15–30 days), increasing BIX values suggested a gradual integration of biologically transformed metabolites into the HA structure. These results further support the dynamic, progressive nature of HA formation. Across all composting stages, HIX values for DOM, FA, and HA remained below 1.5, indicating a relatively low degree of humification. These results suggest that the organic matter in all three fractions remained in an early to intermediate stage of humification, characterized by limited aromatic condensation and the incomplete development of highly stabilized humic structures (Figure 4).

### 2.5. Correlation Between Environmental Factors and Different Composting Ratios of Cow Manure

**Figure 5:**
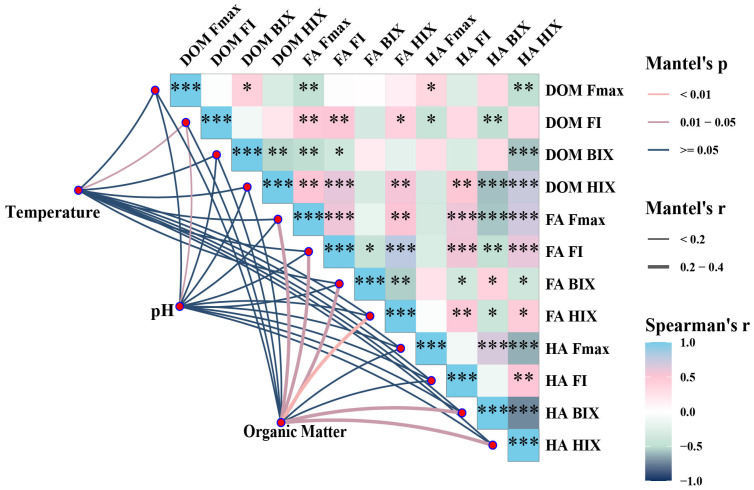
Mantel test analysis of the relationship between fluorescence intensity and fluorescence index under different composting ratios, based on physical and chemical properties. * *p* < 0.05, ** *p* < 0.01, and *** *p* < 0.001.

In the straw–cow manure co-composting system, temperature and pH are the most important environmental variables and largely impact the structural evolution and humification rate of DOM. The Mantel analysis results (Figure 5) indicate that temperature was significantly positively correlated with FA and Fmax (r = 0.9989 ***) (*p* < 0.001), indicating that it is the primary associated with FA formation. The pH showed a significant positive correlation with DOM (r = 0.5514 **) (*p* < 0.01) and a negative correlation with HIX (r = −0.42), suggesting that alkaline conditions inhibit aromatic condensation and HA formation.

**Figure 6:**
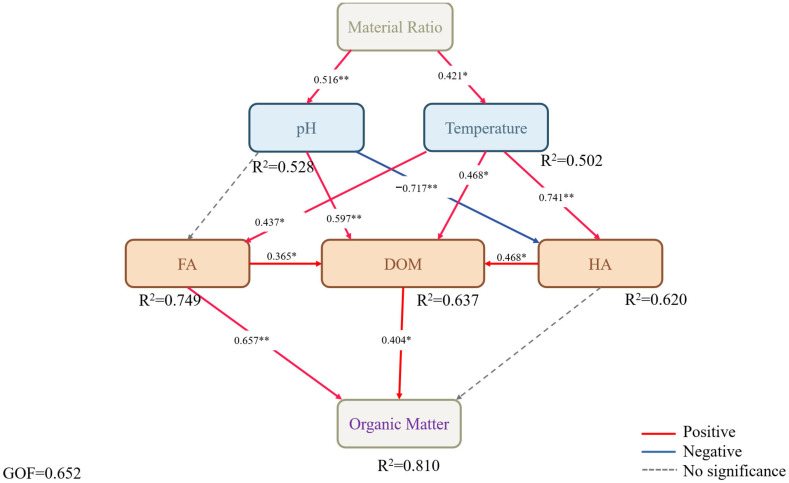
Partial least squares-partial multivariate analysis of fluorescence and organic matter under different composting ratios. * *p* < 0.05, ** *p* < 0.01.

Partial least squares regression (PLS-PM) further revealed the dominant pathways linking environmental factors to DOM components. The model fitting results (GOF = 0.652) indicated that the temperature-to-HA pathway exhibited the highest overall contribution rate (0.74) (Figure 6). This demonstrates that higher temperatures first promote the conversion of DOM into microbial-derived components, subsequently enhance FA aromatization (reflected by increased HIX values), and ultimately drive HA formation and stabilization. Concurrently, the coefficient for the pH-to-HA pathway was −0.72, suggesting that alkaline conditions may constrain condensation-related interactions during humic acid–like substance formation, while higher temperatures are linked to increased microbial activity and the progressive structural complexity of organic matter.

## 3. Discussion

### 3.1. Dynamic Characteristics of DOM Under Different Ratio Treatments

The DOM content in the treatment samples increased first and then decreased [28]. The variation in the Fmax peak of DOM and its timing of emergence during composting directly reveal the central regulatory role of raw material ratios. Treatments with high manure proportions (e.g., T1), rich in soluble organic matter and nitrogen sources, showed rapid early increases in DOM content. This aligns with the initial “rapid mineralization-dominated” phase commonly observed in composting, attributed to the high organic matter content in livestock manure [29]. However, excessive amounts of readily degradable carbon may intensify respiratory losses, causing DOM to be converted into CO_2_ and small-molecule intermediates rather than entering stable humification pathways [30]. Conversely, in treatments with moderate straw ratios (e.g., T2/T3), excessive DOM mineralization was buffered by introducing moderate amounts of structural carbon sources such as lignocellulose. This not only slowed down the DOM degradation rate, resulting in a later peak onset and longer duration, but also provided sustained aromatic skeleton precursors for the accumulation of humic-like components (e.g., C1) [31]. Therefore, treatments with medium ratios achieve moderate enrichment and orderly transformation, with a balance between supplying microorganisms with readily degradable materials and building stable carbon pools. This mechanism is conceptually analogous to introducing refractory materials to enhance humification [32].

### 3.2. Conversion Pathways and Transformation Characteristics of FA and HA

Both FA and HA are active intermediates and stable end products, respectively, in the humification process. Analysis of the relative content Fmax revealed that the temporal and structural differences observed in this study reflect a reconfiguration of humification pathways under raw material ratio regulation. In the medium cow manure and higher straw ratio treatments, FA rapidly accumulated during the early to mid-composting stages and progressively underwent aromatization. Conversely, HA showed significant increases in the mid-to-late stages, accompanied by a heightened fluorescence intensity and elevated HIX values. This indicates that a portion of FA was irreversibly converted to HA via condensation and oxidative polymerization [33]. This reflects a continuous process during the composting cycle: rapid generation of easily degradable components, sustained enrichment of aromatic structures during the mid-composting phase, and the gradual accumulation of high-molecular-weight humic substances in the late composting stage. This result aligns with the observed trend of FA to HA structural transformation in composting [34]. From an environmental perspective, moderately accumulated and structurally mature FA enhances the system’s capacity to chelate nutrients and trace heavy metals. A higher HA content and aromaticity directly determine the carbon sequestration potential and long-term stability of compost products [35]. In the present study, a moderate ratio (e.g., T2/T3) avoids both the “excessive active DOM” and potential environmental risks caused by the long-term high-level retention of FA while significantly promoting HA accumulation and structural aging. This demonstrates that optimizing the ratio of straw and cattle manure to regulate the flux and efficiency of FA conversion to HA achieves synergistic effects between short-term nutrient availability and long-term carbon stability.

### 3.3. Mechanism Underlying the Regulation of Organic Matter Transformation Pathways by Raw Material Ratios

Based on the FI analysis, the DOM sources during composting exhibit dual characteristics: FA predominantly originates from microbial sources, whereas HA undergoes a dynamic transition from primarily substrate-derived to a combination of both substrate-derived and endogenous sources. The BIX results reveal that the autogenic contribution to DOM gradually diminishes as composting progresses. The FA maintains a high degree of autogenic characteristics, whereas HA exhibits enhanced autochthonous origin in the later stages due to the incorporation of microbial metabolites [36]. The overall low HIX values indicate that under the conditions established in this study, the humification of the material remains in its initial stage. Organic matter predominantly consists of biologically active components that have not yet entered the advanced humification stage [37]. During the high-ratio composting of cattle manure, easily degradable carbon and nitrogen dominate, strongly stimulating microbial activity and promoting rapid organic matter mineralization. This manifests as a sudden onset of the high-temperature phase, an elevated early peak in dissolved organic matter (DOM), and a high proportion of protein-like components with limited humification (HIX) [38]. Although this process proceeds rapidly, it carries a high risk of carbon and nitrogen loss, resulting in the insufficient accumulation of stable humus. During composting with a high straw proportion, structural carbon predominates, leading to a high C/N ratio. Initially, microbial activity is nitrogen-limited, causing a slow temperature rise and weak mineralization. Although the losses are reduced, limited microbial activity and nitrogen sources may impede the condensation of aromatic precursors into HA, leading to a slowdown or even stagnation of humification [39]. By uniformly adding urea to regulate the initial C/N ratio, it may have a transient impact on the composition of dissolved organic matter, microbial activity, and their fluorescence characteristics during the early stage of composting [40]. As a readily available inorganic nitrogen source, urea can rapidly alleviate the initial nitrogen limitation and enhance microbial growth and enzymatic activity, thereby accelerating the decomposition process of easily degradable organic substrates, and transiently enhance the microbial contribution to DOM as reflected in elevated protein-like fluorescence signals [41]. However, urea itself is not a structural precursor of fluorescent humus; it rapidly hydrolyzes in the system and is assimilated and utilized by microorganisms, rather than directly participating in the construction of DOM or humus structure. Based on the results of this study, the medium-ratio treatment successfully combined the advantages of both raw materials: the easily degradable components and nitrogen source provided by cattle manure ensured microbial metabolic activity, driving initial decomposition and FA production, whilst the structural carbon from straw served as a physical scaffold and chemical reaction substrate, prolonging the retention time of organic matter and supplying precursors for FA aromatization and polymerization into HA. This synergistic model of “active substrate-associated with structural framework-supported” processes shares the same principle as the addition of biochar or mineral materials to optimize the composting microenvironment and promote humification, establishing an environment conducive to sustained microbial relay transformations and chemical condensation reactions [42]. Future studies incorporating longer composting periods and complementary structural characterization techniques are required to further elucidate the progression toward mature humic substances.

### 3.4. Environmental Factors Affecting Humification and the Underlying Mechanisms

The Mantel analysis and PLS-PM model results indicate that temperature is the main environmental factor driving humus formation, whereas pH regulates the composting process. Temperature primarily promotes microbial metabolism, increasing the yield of protein-like components and microbial-derived humus (C3) (manifested as initial increases in FI and BIX). It also provides sustained thermodynamic energy for subsequent aromatic condensation reactions, promoting HA formation and stabilization. This observation is consistent with previous studies showing that elevated temperatures facilitate the progressive transformation of lignin degradation products into more structurally complex forms [43,44]. However, whilst a slightly alkaline environment (increased pH) promoted initial nitrogen retention, it also inhibited HA formation (the pH-to-HA pathway coefficient is negative). This may be attributed to the fact that alkaline conditions impede key reactions, indicating that changes in the aromaticity and structural complexity of organic matter under different composting conditions are associated with progressive microbial transformation and reorganization processes, rather than being attributed to a specific condensation reaction between functional groups [45]. In this sense, the transition from slightly alkaline to neutral-to-acidic conditions may be most conducive to the full progression of humification: the initial slightly alkaline phase promotes ammonia nitrogen preservation and microbial activity, whereas the subsequent acidic conditions facilitate condensation reactions [46]. Our findings demonstrate that a straw-to-manure ratio of 6:4 at T3 can achieve a balanced trade-off between composting efficiency and product stability, making it more suitable for field applications requiring both nutrient provision and long-term organic matter persistence. This quantitative analysis highlights the practical significance of optimizing raw material ratios in sustainable compost production. A high manure proportion enhances microbial activity and composting efficiency, while a high straw proportion promotes aromatic structure formation and the potential for humus formation. In this study, we quantified this continuous regulatory process for the first time in a straw–cow manure composting system using a pathway model, demonstrating that temperature associated with microbial–chemical coupling processes is central to humus quality formation. Humification is defined as the overall directional transformation of compost organic matter toward chemically more complex and relatively stabilized structures. Polymerization and polycondensation are considered possible contributing reaction pathways within this broader process, particularly involving phenolic and quinone-type intermediates. The formation of condensed aromatic structures represents one structural manifestation of increasing molecular complexity and stabilization, but should not be regarded as synonymous with humification itself. This distinction is consistent with contemporary models of humic substance evolution.

## 4. Materials and Methods

### 4.1. Experimental Set Up and Sample Collection

The experimental site was established at the drying yard of the Heilongjiang Academy of Agricultural Sciences in Harbin City, Heilongjiang Province (45°69′ N, 126°63′ E), with good ventilation conditions. Composting was conducted in a small plastic insulated box (80 L) that could be tightly sealed, and the lid could be opened for turning and ventilation. Four treatments were established, with corn stover and cattle manure mixed at the following volume ratios: T1 (2:8), T2 (4:6), T3 (6:4), and T4 (8:2), with three replicates per treatment. The materials were thoroughly mixed, and urea was uniformly added to adjust the initial C:N ratio to 25:1, thereby minimizing differences in nitrogen availability among treatments. In this study, the potential influence of urea is considered to be indirect and biological rather than analytical in nature. It is rapidly hydrolyzed and assimilated by microorganisms. Consequently, urea does not directly interfere with excitation–emission matrix (EEM) measurements or the calculation of FI, BIX, and PARAFAC-resolved component loadings, maintaining the moisture content at approximately 55–60%. During the experiment, the pile was turned once every 4 days using a shovel, and the composting period was 40 days.

Samples were collected on Days 0, 10, 15, 20, 25, 30, 35, and 40 during composting. During sampling, 50 g of material was taken from the upper, middle, and lower sections of the bin, respectively, with three replicates per section, and thoroughly mixed. A total of 96 excitation–emission matrix (EEM) fluorescence datasets (4 treatments × 8 sampling times × 3 replicates) were included in the PARAFAC modeling. The samples were air-dried, pulverized, sieved through a 2 mm mesh, and stored at room temperature (15–20 °C).

### 4.2. DOM Extraction and Spectral Analysis

For DOM extraction, 10 g of fermented and air-dried sample was weighed and transferred into a 50 mL centrifuge tube. Subsequently, we added 30 mL of ultrapure water (Milli-Q water) at a water-to-fertilizer ratio of 1:5 (mass-to-volume ratio). The mixture was incubated in the dark at 25 °C and 200 rpm for 12 h [47]. After shaking, the suspension was centrifuged at 25 °C and 3500 rpm for 15 min (Sorvall ST 16R, Thermo Fisher Scientific, Germany), and the obtained supernatant was filtered through a 0.45 μm cellulose membrane [48]. The samples were stored in the dark at 4 °C for subsequent analyses.

For FA and HA extraction, 10 g of compost was mixed with 30 mL of 0.1 mol/L NaOH and 0.1 mol/L Na_4_P_2_O_7_ (pH = 13) solution, and the extraction was performed by shaking (180 rpm, 25 °C) for 24 h. Subsequently, the mixture was centrifuged at 4500 rpm for 15 min, the supernatant was collected, and the solution was filtered through a 0.45 μm fiber resin membrane. The pH of the filtrate was adjusted to 1.0 using 6 mol/L HCl, and the filtrate was kept at room temperature for 12 h, followed by centrifugation at 4500 rpm for 15 min. The supernatant and precipitate were collected, and the supernatant was filtered to obtain FA. The precipitate was dissolved using 0.05 mol/L NaHCO_3_ and diluted to 50 mL to obtain HA.

We used a fluorescence spectrometer Model F-7000 (Hitachi High-Tech Corporation, Japan) with the following specifications: excitation source 450 W xenon arc lamp; PMT voltage 700 V; signal-to-noise ratio > 110; bandpass Ex = 5 nm, Em = 5 nm; scan speed 1200 nm min^−1^; response time automatic instrument calibration during spectral scanning. The three-dimensional fluorescence spectral scanning range was Ex = 200–600 nm/Em = 200–600 nm. To prevent secondary Rayleigh scattering in the fluorescence spectra, a 290 nm cutoff filter was added on the fluorescence emission side. Data were collected using the instrument’s software (FLWinLab software Version 2.1.0, PerkinElmer, 940 Winter Street, Waltham, MA, USA). For parallel factor analysis, ultrapure water served as the blank as it exerts a relatively higher influence. All spectral data underwent internal filter effect correction by subtracting ultrapure water blanks and integrating the corresponding absorbance data.

### 4.3. Statistical Analysis

One-way analysis of variance (ANOVA) was employed to assess the significance of differences among treatments at varying sampling days (*p* < 0.05). The correlation analyses between different sampling days and maximum fluorescence intensity, as well as fluorescence indices, were performed using Origin 2024b and Prism 10.6. Additionally, partial least squares path modeling (PLS-PM) was implemented via the “plspm” package in R software (version 4.5.2), and Mantel tests were conducted to examine the correlations between physicochemical parameters and fluorescence indices/intensities using the “Mantel test” package. Data visualization and correlation analysis were performed in the software packages SPSS 19.0, Origin 2024b, and Prism 10.6. Fluorescence spectral data analysis employed the MATLAB R2024a software integrated with the DOMFluor toolbox (http://www.models.life.ku.dk, accessed on 21 October 2025) to conduct parallel factor analysis (PARAFAC) on three-dimensional fluorescence data for DOM, FA, and HA across all experimental samples. After outlier removal, the optimal number of components was validated through residual analysis and split-half analysis. The identified fluorescent components were compared with reference spectra in the OpenFluor database (https://openfluor.lablicate.com/, accessed on 21 October 2025) to verify the analysis accuracy. The relative content of each component was expressed as its maximum fluorescence intensity (Fmax, relative units: R.U.).

The fluorescence index (FI) was calculated using the ratio of fluorescence intensity at emission wavelengths of 450 and 500 nm at an excitation of 370 nm [49]. The calculation method for the biotic index (BIX) is as follows: the ratio of fluorescence intensity at emission wavelengths of 380 and 430 nm at an excitation of 310 nm [50]. The humification index (HIX) is defined as the ratio of the integrated fluorescence intensity in the 435–480-nm emission wavelength range to that in the 300–345-nm range, measured at an excitation wavelength of 254 nm [51].

Although the experiments were conducted in an 80 L controlled composting system, the primary objective of this study was to elucidate relative differences and directional trends in DOM evolution and humification potential among different straw–cattle manure ratios, rather than to quantify absolute composting rates. The controlled conditions enabled the isolation of material composition effects, which are fundamentally relevant across composting scales. While larger-scale systems may exhibit increased heterogeneity in temperature, aeration, and moisture, the comparative influence of raw material ratios on composting efficiency and humification tendencies is expected to be transferable within a reasonable operational range.

## 5. Conclusions

We systematically analyzed the fluorescence characteristics of DOM, FA, and HA during composting under different straw–cow manure ratios, along with their responses to various environmental factors. Varying material ratios influenced the heating rates, the duration of the high-temperature phase, and the pH dynamics, thereby altering the decomposition, accumulation, and transformation pathways of organic matter. A moderate ratio (T2/T3) promoted moderate enrichment and the orderly transformation of organic components within DOM while maintaining suitable microbial activity. The compounds DOM, FA, and HA contained 3, 2, and 3 types of fluorescent components, respectively, including humic-like, protein-like, and microbial-derived components. The optimal ratio facilitates the accumulation of humic-like components and the structural conversion of FA to HA, facilitating humification. Fluorescence index analysis indicated that DOM has dual origins: FA represents a typical microbial source, whilst HA transitions from a substrate-derived to a mixed substrate-derived-endogenous type. The BIX shows that the autogenic contribution of DOM diminishes with composting, FA maintains a consistently high autogenicity, and HA exhibits an enhanced autogenicity in the later stages due to the incorporation of microbial metabolites. The overall HIX < 1.5 reflects that under the study conditions, humification remained in the primary stage dominated by biological sources. Temperature was the main factor associated with humic acid (HA) formation and stabilization, promoting humification through microbial–chemical coupling. Alkaline conditions inhibit humic condensation reactions, whereas pH dynamics influence the final humic structure. The medium straw–cow manure ratio maintained microbial metabolic activity by balancing inputs of readily degradable carbon and structural carbon while providing sustained substrates and structural support for aromatization and polymerization. In summary, the medium-level raw material ratio significantly influenced organic matter transformation pathways and humification by regulating the microenvironment. The results of this study serve as a theoretical foundation for optimizing material ratios in agricultural waste resource use. Longer-term composting experiments and complementary structural analyses are therefore required to further evaluate the formation and stabilization of mature humic substances.

## Data Availability

Data are contained within the article.

## Abbreviations

The following abbreviations are used in this manuscript:

DOM	Dissolved organic matter
FA	Fulvic acid
HA	Humic acid
FI	Fluorescence index
BIX	Biotic index
HIX	Humification index

## References

[1] Bian B., Hu X., Zhang S., Lv C., Yang Z., Yang W., Zhang L. (2019). Pilot-scale Composting of Typical Multiple Agricultural Wastes: Parameter Optimization and Mechanisms. Bioresour. Technol..

[2] Chen L., Chen Y., Li Y., Liu Y., Jiang H., Li H., Yuan Y., Chen Y., Zou B. (2023). Improving the Humification by Additives during Composting: A Review. Waste Manag..

[3] Jiao Z., Li R., Zhang K., Zhang Y., Guo Y., Chang S., Chang Y., Wei Y., Kang Z., Qiao Y. (2025). From Carbon Sequestration Perspective: Adsorption of Minerals Enhances the Stabilization of Organic Fractions in Composting. Environ. Technol. Innov..

[4] Chen L., Zhang Z., Yang R., Wang X., Yu J., Jiang H., Zhang W., Xi B., Sun X., Li N. (2024). Nano Fe_3_O_4_ improved the electron donating capacity of dissolved organic matter during sludge composting. J. Environ. Manag..

[5] Pizzanelli S., Calucci L., Forte C., Borsacchi S. (2023). Studies of Organic Matter in Composting, Vermicomposting, and Anaerobic Digestion by 13C Solid-State NMR Spectroscopy. Appl. Sci..

[6] Piccolo A. (2016). In memoriam Prof. F.J. Stevenson and the Question of Humic Substances in Soil. Chem. Biol. Technol. Agric..

[7] Gerke J. (2018). Concepts and Misconceptions of Humic Substances as the Stable Part of Soil Organic Matter: A Review. Agronomy.

[8] Fuentes M., González-Gaitano G., García-Mina J.M. (2006). The Usefulness of UV–Visible and Fluorescence Spectroscopies to Study the Chemical Nature of Humic Substances from Soils and Composts. Org. Geochem..

[9] Zhang F., Li Y., Xiong X., Yang M., Li W. (2012). Effect of composting on dissolved organic matter in animal manure and its binding with Cu. Sci. World J..

[10] Lanno M., Klavins M., Purmalis O., Shanskiy M., Kisand A., Kriipsalu M. (2022). Properties of humic substances in composts comprised of different organic source material. Agriculture.

[11] Stedmon C.A., Bro R. (2008). Characterizing Dissolved Organic Matter Fluorescence with Parallel Factor Analysis: A Tutorial. Limnol. Oceanogr. Methods.

[12] Yu H., Luo H., Wu J., Tang Z., Liu Y., Yang M., Shen R. (2010). PARAFAC Modeling of Fluorescence Excitation–Emission Spectra for Rapid Assessment of Compost Maturity. Bioresour. Technol..

[13] Yu Z., Liu X., Zhao M., Zhao W., Liu J., Tang J., Liao H., Chen Z., Zhou S. (2019). Hyperthermophilic Composting Accelerates the Humification Process of Sewage Sludge: Molecular Characterization of Dissolved Organic Matter Using EEM–PARAFAC and Two-Dimensional Correlation Spectroscopy. Bioresour. Technol..

[14] Liu D., Nie L., Xi B., Gao H., Yang F., Yu H. (2024). A Novel Approach for Identifying Sources of Fluvial DOM Using Fluorescence Spectroscopy and Machine Learning Model. npj Clean Water.

[15] Shi F., Xu C., Liu J., Sun F., Yu H., Wang S., Li P., Yu Q., Li D., Zuo X. (2022). Static composting of cow manure and corn stalk covered with a membrane in cold regions. Front. Bioeng. Biotechnol..

[16] Wei J., Shangguan H., Shen C., Mi H., Liu X., Fu T., Tang J., Zhou S. (2022). Deciphering the Structural Characteristics and Molecular Transformation of Dissolved Organic Matter during the Electrolytic Oxygen Aerobic Composting Process. Sci. Total Environ..

[17] Arcus V.L., Prentice E.J., Hobbs J.K., Mulholland A.J., van der Kamp M.W., Pudney C.R., Parker E.J., Schipper L.A. (2016). On the Temperature Dependence of Enzyme-Catalyzed Rates. Biochemistry.

[18] Yang X., Zhang J., Mostofa K.M.G., Mohinuzzaman M., Teng H.H., Senesi N., Senesi G.S., Yuan J., Liu Y., Li S.-L. (2025). Solubility Characteristics of Soil Humic Substances as a Function of pH: Mechanisms and Biogeochemical Perspectives. Biogeosciences.

[19] Liu Q., He X., Wang K., Li D. (2023). Biochar Associated withs Humus Formation during Composting by Regulating the Specialized Metabolic Features of Microbiome. Chem. Eng. J..

[20] Jiang J., Wang Y., Yu D., Hou R., Ma X., Liu J., Cao Z., Cheng K., Yan G., Zhang C. (2022). Combined Addition of Biochar and Garbage Enzyme Improving the Humification and Succession of Fungal Community during Sewage Sludge Composting. Bioresour. Technol..

[21] Huang X., Fu X., Zhao Z., Yin H. (2024). The Telltale Fluorescence Fingerprints of Sewer Flows for Interpreting the Low Influent Concentration in Wastewater Treatment Plant. J. Environ. Manag..

[22] Logozzo L.A., Hosen J.D., McArthur J., Raymond P.A. (2023). Distinct Associated withrs of Two Size Fractions of Operationally Dissolved Iron in a Temperate River. Limnol. Oceanogr..

[23] Pucher M., Flödl P., Graeber D., Felsenstein K., Hein T., Weigelhofer G. (2021). Complex Interactions of In-Stream Dissolved Organic Matter and Nutrient Spiralling Unravelled by Bayesian Regression Analysis. Biogeosciences.

[24] Hong H., Wu S., Wang Q., Dai M., Qian L., Zhu H., Li J., Zhang J., Liu J., Li J. (2021). Fluorescent Dissolved Organic Matter Facilitates the Phytoavailability of Copper in the Coastal Wetlands Influenced by Artificial Topography. Sci. Total Environ..

[25] Meilleur C., Kamula M., Kuzyk Z.A., Guéguen C. (2023). Insights into Surface Circulation and Mixing in James Bay and Hudson Bay from Dissolved Organic Matter Optical Properties. J. Mar. Syst..

[26] Amaral V., Ortega T., Romera-Castillo C., Forja J. (2021). Linkages between Greenhouse Gases (CO_2_, CH_4_, and N_2_O) and Dissolved Organic Matter Composition in a Shallow Estuary. Sci. Total Environ..

[27] Grunert B.K., Tzortziou M., Neale P., Menendez A., Hernes P.J. (2021). DOM Degradation by Light and Microbes along the Yukon River–Coastal Ocean Continuum. Sci. Rep..

[28] Maurischat P., Lehnert L., Zerres V.H.D., Tran T.V., Kalbitz K., Rinnan Å., Li X.G., Dorji T., Guggenberger G. (2022). The Glacial–Terrestrial–Fluvial Pathway: A Multiparametrical Analysis of Spatiotemporal Dissolved Organic Matter Variation in Three Catchments of Lake Nam Co, Tibetan Plateau. Sci. Total Environ..

[29] Gong B., Zhong X., Chen X., Li S., Hong J., Mao X., Liao Z. (2021). Manipulation of Composting Oxygen Supply to Facilitate Dissolved Organic Matter (DOM) Accumulation Which Can Enhance Maize Growth. Chemosphere.

[30] Zhang Z., Yang H., Linghu M., Li J., Chen C., Wang B. (2024). Cattle Manure Composting Associated withn by a Microbial Agent: A Coupled Mechanism Involving Microbial Community Succession and Organic Matter Conversion. Sci. Total Environ..

[31] Ye P., Fang L., Song D., Zhang M., Li R., Awasthi M.K., Zhang Z., Xiao R., Chen X. (2023). Insights into Carbon Loss Reduction during Aerobic Composting of Organic Solid Waste: A Meta-Analysis and Comprehensive Literature Review. Sci. Total Environ..

[32] Li H., Liu C., Luo X., Zhuo G., Zheng Y., Zhen G. (2025). Enhancing Kitchen Waste Composting by Cellulolytic Microorganisms: New Insights from Quorum Sensing and Carbohydrates Metabolic Functions. Chem. Eng. J..

[33] Jiao M., Ren X., He Y., Wang J., Hu C., Zhang Z. (2023). Humification Improvement by Optimizing Particle Size of Bulking Agent and Relevant Mechanisms during Swine Manure Composting. Bioresour. Technol..

[34] Xie T., Zhang Z., Yu Y., Tian Y., Wang F., Li D., Nan J., Feng Y. (2023). Aeration Intensity Associated withs Dissolved Organic Matter Transformation and Humification during Composting by Regulating the Organics Metabolic Functions of Microbiome. Chem. Eng. J..

[35] Wang Y., Wei Y., Zhou K., Gao X., Chang Y., Zhang K., Deng J., Zhan Y., Li J., Li R. (2023). Regulating pH and *Phanerochaete chrysosporium* inoculation improved the humification and succession of fungal community at the cooling stage of composting. Bioresour. Technol..

[36] Leno N., Ajayan A.S., Thampatti K.C.M., Sudharmaidevi C.R., Aparna B., Gladis R., Rani T.S., Joseph B., Meera A.V., Nagula S. (2022). Humification Evaluation and Carbon Recalcitrance of a Rapid Thermochemical Digestate Fertiliser from Degradable Solid Waste for Climate Change Mitigation in the Tropics. Sci. Total Environ..

[37] Bai B., Liu H., Liang A., Wang L., Wang A. (2025). Linking fluorescence spectral to machine learning predicts the emissions fates of greenhouse gas during composting. Comput. Electron. Agric..

[38] Kwiatkowska-Malina J. (2018). Qualitative and Quantitative Soil Organic Matter Estimation for Sustainable Soil Management. J. Soils Sediments.

[39] Chang T., Lee H., Hsieh Y., Chen C., Jien H. (2023). Using Fluorescence Spectroscopy to Assess Compost Maturity Degree during Composting. Agronomy.

[40] Li Y., Li J., Chang Y., Li R., Zhou K., Zhan Y., Wei R., Wei Y. (2023). Comparing Bacterial Dynamics for the Conversion of Organics and Humus Components during Manure Composting from Different Sources. Front. Microbiol..

[41] Staley C., Breuillin-Sessoms F., Wang P., Kaiser T., Venterea R.T., Sadowsky M.J. (2018). Urea Amendment Decreases Microbial Diversity and Selects for Specific Nitrifying Strains in Eight Contrasting Agricultural Soils. Front. Microbiol..

[42] Qiao N., Xu X., Hu Y., Blagodatskaya E., Liu Y., Schaefer D., Kuzyakov Y. (2016). Carbon and Nitrogen Additions Induce Distinct Priming Effects along an Organic-Matter Decay Continuum. Sci. Rep..

[43] Gupta S., Yildirim S., Andrikopoulos B., Wille U., Roessner U. (2023). Deciphering the Interactions in the Root–Soil Nexus Caused by Urease and Nitrification Inhibitors: A Review. Agronomy.

[44] Qiao X., Li P., Zhao J., Li Z., Li Z., Zhang C., Wu J. (2024). Gaining insight into the effect of laccase expression on humic substance formation during lignocellulosic biomass composting. Sci. Total Environ..

[45] Cao Y., Gu J., Zhang J., Chen B., Xu Y., Liu D., Hu H., Huang H. (2022). Reduced pH Is the Primary Factor Promoting Humic Acid Formation during Hyperthermophilic Pretreatment Composting. J. Environ. Manag..

[46] Xu M., Wei Y., Ma S., Zhou H., Xu M., Zhan Y. (2025). Neutral Initial pH Enhances the Formation of Humic Acid by Inhibiting the Growth of *Lactobacillus* in Food Waste Composting. Environ. Technol. Innov..

[47] Wang X.X., Zhou L.Y., Fu Y.L., Jiang Z., Jia S.X., Song B.Q., Liu D.Q., Zhou X.H. (2024). Drought-Induced Changes in Rare Microbial Community Promoted Contribution of Microbial Necromass C to SOC in a Subtropical Forest. Soil Biol. Biochem..

[48] Zhao B., Wang Y., Li L., Ma L., Deng Y., Xu Z. (2023). Adjusting pH of the Secondary Composting Materials to Further Enhance the Lignocellulose Degradation and Promote the Humification Process. Sustainability.

[49] McKnight D.M., Boyer E.W., Westerhoff P.K., Doran P.T., Kulbe T., Andersen D.T. (2001). Spectrofluorometric Characterization of Dissolved Organic Matter for Indication of Precursor Organic Material and Aromaticity. Limnol. Oceanogr..

[50] Huguet A., Vacher L., Relexans S., Saubusse S., Froidefond J.-M., Parlanti E. (2009). Properties of Fluorescent Dissolved Organic Matter in the Gironde Estuary. Org. Geochem..

[51] Zsolnay A., Baigar E., Jimenez M., Steinweg B., Saccomandi F. (1999). Differentiating with Fluorescence Spectroscopy the Sources of Dissolved Organic Matter in Soils Subjected to Drying. Chemosphere.

